# How do we reimagine health in a digital age?

**DOI:** 10.2471/BLT.19.235358

**Published:** 2020-04-01

**Authors:** Flavia Bustreo, Marcel Tanner

**Affiliations:** a Fondation Botnar, St. Alban-Vorstadt 56, 4052 Basel, Switzerland.

Digital technologies, such as artificial intelligence, offer innovative solutions to some of the challenges of the sustainable development goals, particularly in resource-constrained settings.^1^^,^^2^ Digital solutions and artificial intelligence have the potential to transform health care and improve access to services by addressing financial, social or geographic factors. The digital future appears positive; however, we must also consider new ethical and human rights risks arising from the application of these technological developments in public health.^3^^,^^4^

The benefits of big data should not come at the expense of data privacy rights or be used for purposes other than those originally intended.^5^ We recognize the range of ethical issues related to the collection, sharing, repurposing and misuse of big data, and acknowledge that biased and unrepresentative data can contribute to increasing the gap between those who benefit and those who are left behind.^6^ Making sure these concerns are addressed through better regulation and mechanisms of accountability is critical. Collaboration between private and public-sector actors is essential to achieve this goal, and Fondation Botnar aims to facilitate and mobilize these partnerships.

The key to preventing a group from monopolizing power and wealth is the regulation of data ownership. Groups could use collected data for commercial gain; therefore, we must act now to avoid this risk by working with partners to advocate for the creation of a global governance framework, ensuring that health data becomes a global public good.^6^

Digital innovations for health are not always driven by those who should ultimately benefit or who have expertise in conducting ethical and equitable public health research and programmes.^7^ This issue has contributed to the failure of many digital health solutions to address health disparities.^8^ For example, women, children and adolescents in resource-constrained settings need more accessible and affordable quality care, yet despite the expanding digital space, few innovations specifically target women, children and adolescents.^9^ In addition to gender bias in health-care technologies, a gender divide in access to and affordability of digital tools also exists.^10^ The poorest and most vulnerable groups must also benefit from the digital era and should participate in the innovation process as decision-makers and beneficiaries of digital tools through participatory user-led and user-centred digital health design processes.^7^^,^^10^

Through the Fondation Botnar programmes, we support dignified and fair technologies, that is, digital technologies that go beyond involving people as commercial users, and instead place people’s needs and empowerment at the centre of proposed solutions. We believe technology should promote, rather than diminish health equity gains, by focusing on the underserved. For example, an artificial-intelligence enabled solution, in the form of a health mobile application, is enabling women, children and adolescents living in areas with limited access to health care in Romania and the United Republic of Tanzania, to obtain personalized and high-quality health guidance in their local language, for their physical and mental health.^11^

Bridging the digital divide is key to leveraging the benefits of these new tools, and we must find new and innovative ways to reach those who still do not have access to the internet. The next generation of digital natives must lead this transformation: young people must drive the agenda on the role of technology in their health and well-being.^12^

We welcome the contribution of the theme issue on the new ethical challenges of digital technologies, machine learning and artificial intelligence in public health of the *Bulletin of the World Health Organization* to tackling many of the key ethical challenges of digital health. New technologies pose challenges for public health ethics; however, they also present an opportunity to develop health ethics for digital technologies.

At Fondation Botnar, we strive to reimagine health in a digital age that is inclusive of all groups, starting from the most vulnerable young women and adolescents.
